# Establishment and validation of risk prediction model to predict intravenous immunoglobulin-resistance in Kawasaki disease based on meta-analysis of 15 cohorts

**DOI:** 10.1186/s13052-025-01889-w

**Published:** 2025-02-21

**Authors:** Shuhui Wang, Na Sun, PanPan Liu, Weiguo Qian, Qiuqin Xu, DaoPing Yang, Mingyang Zhang, Miao Hou, Ye Chen, Guanghui Qian, Chunmei Gao, Ling Sun, Haitao Lv

**Affiliations:** 1 Department of Cardiology, Children’s Hospital of Soochow University, No 92, Zhong-nan Street, Suzhou, Jiangsu 215003 China; 2 Department of Health Statistics, School of Public Health, Shandong Second Medical University, Weifang, Shandong 261053 China

**Keywords:** Kawasaki disease, Intravenous immunoglobulin, Risk factor, Prediction model

## Abstract

**Background:**

Pediatric Kawasaki disease (KD) patients showing resistance to intravenous immunoglobulin (IVIG) are at risk of coronary artery lesions; thus, early prediction of IVIG resistance is particularly important. Herein, we aimed to develop and verify a novel predictive risk model for IVIG resistance in KD based on meta-analyses.

**Methods:**

PubMed, Embase, and Web of Science databases were searched for cohort studies on the risk factors for IVIG resistance from January 2006 to December 2022. Data were extracted from the screened literature, followed by quality assessment using the Newcastle-Ottawa scale. meta-analyses used Stata 17.0 software to extract the risk factors with significant combined effect sizes and combined risk values, followed by logistic regression prediction model construction. The model was prospective validated using data from 1007 pediatric KD cases attending the Children’s Hospital of Soochow University. The model’s predictive ability was assessed using the Hosmer–Lemeshow test and area under the receiver operating characteristic curve (AUC) and the clinical utility was assessed using decision curve analysis(DCA).

**Results:**

Fifteen cohort studies reporting 4273 patients with IVIG resistance were included. The incidence of IVIG resistance was 16.2%. Six risk factors were reported ≥ 3 times with significant results for the combined effect size: male sex, rash, cervical lymphadenopathy, % neutrophils ≥ 80%, Age ≤ 12 months and platelet count ≤ 300 × 10^9^/L. The logistic scoring model had 83.8% specificity, 70.4% sensitivity, and an optimal cut-off value of 23.500.

**Conclusion:**

The risk prediction model for IVIG resistance in KD showed a good predictive performance, and pediatricians should pay high attention to these high-risk patients and develop an appropriate individual regimens to prevent coronary complications.

**Supplementary Information:**

The online version contains supplementary material available at 10.1186/s13052-025-01889-w.

## Highlights

Accurate identification of IVIG-resistant patients in the early course of Kawasaki disease (KD) allows for early and timely intervention and the prevention of occurrence of CALs;

Based on meta-analyses, logistic regression was employed to develop and validate a risk prediction model for IVIG resistance in KD, which proved to have good predictive power and provided new ideas for model establishment.

This study is the first to establish a prediction model for IVIG resistance in KD based on high-quality cohort studies and meta-analyses, which not only avoids missing data, but also ensures sufficient sample size to compensate for the instability of previous prediction models.

**Supplementary Information:**

The online version contains supplementary material available at 10.1186/s13052-025-01889-w.

## Introduction

The immune vasculitis disorder, Kawasaki disease (KD, or cutaneous mucosal lymph node syndrome), occurs frequently in children. This disease tends to affect infants, males, and Asian populations, and is currently considered to be the primary cause of acquired heart disease in children [1]. KD is frequently complicated by coronary artery lesions (CALs), and the damage of coronary endothelial cells is always throughout the whole course of the disease, which is closely related to long-term damage to the heart [2, 3].

Early high-dose intravenous immunoglobulin (IVIG) combined with aspirin treatment can reduce the risk of CALs in patients with KD; however, 7.5–26.8% of patients do not respond to the initial IVIG treatment [4]. IVIG-resistant patients have an increased risk of coronary artery aneurysm (CAA), and might suffer serious life-threatening complications, especially in children. Therefore, early identification and additional appropriate intervention in high-risk children can reduce the probability of coronary artery injury, and such patients might benefit from more aggressive treatment from the beginning of the disease course.

Exploration and early identification of risk factors for IVIG-resistance in KD have been the primary focus of researchers both domestically and internationally. Despite numerous studies establishing risk prediction models for IVIG-resistant KD, there remains a lack of universally recognized and standardized models suitable for clinical application worldwide [5, 6]. Analysis of existing models reveals variations in the inclusion of risk factor variables among different prediction models, with most relying on retrospective data, limited sample sizes, absence of internal and external validation, as well as selection bias, leading to inadequate model stability [7].

Therefore, we carried out meta-analyses to evaluate and integrate the risk factors of IVIG-resistant KD with a significant combined effect size, expanded the sample size of the model, constructed an evidence-based risk prediction model, and subjected it to external validation. Our aim is achieving early identification and timely and effective intervention for the high-risk population with IVIG-resistant KD through early prediction, which is an effective strategy to prevent CALs in KD.

## Methods

### Meta-analyses

#### Literature search

We systematically searched the literature collected in the Web of science, Embase, and PubMed databases (January 2006 to December 2021). The following search terms were used: [“Mucocutaneous lymph node syndrome” OR “Kawasaki disease” OR “Kawasaki Syndrome”] AND [“IVIG resistance” OR “IVIG non-responsiveness” OR “IVIG unresponsiveness”] AND [“predict” OR “score” OR “nomogram” OR “model” OR “risk factor”]. The literature search details of the strategy are presented in Supplementary Table 1. At the same time, the references of the included literature and similar studies were searched manually to supplement the search results. Two reviewers conducted independent literature searches and analysis. The protocol was registered on PROSPERO (Prospero registration number: CRD42024611806). To accurately present this meta-analysis, we followed the PRISMA (Preferred Reporting Program for Systematic Reviews and meta-analyses) guidelines for comprehensive reporting of systematic reviews and meta-analyses.

#### Eligibility criteria

We included all reported model development studies on IVIG-resistant Kawasaki disease. Table 1 shows a detailed description of the PICOTS for the review. Inclusion criteria: (1) A cohort study in English; (2) Children that met the diagnostic criteria of KD or common criteria of the American Heart Association (AHA), and all children were treated with regular dose (2 g/kg × 1 d or 1 g/kg × 2 d) of IVIG. (3) At least one well-defined risk factor variable for non-response to immunoglobulin in KD was reported; the effect value of multivariate risk factor variables, such as the odds ratio (OR) and the 95% confidence interval (95% CI), were reported. (4) A clear description of the statistical methods and correct statistical analysis. The following types of studies were excluded: (1) Basic research literature; (2) reviews, case reports, and meta-analyses; (3) studies whose results could not be translated into OR and 95% CI data; (4) studies whose outcomes did not merely include IVIG resistance.

**Table 1:** Key items for framing the aim, search strategy, and study inclusion and exclusion criteria for review, in accordance with the PICOTS guidance

Item	Definition
Population	Patients with KD were identified
Intervention	The model was prospectively used to predict non-response to IVIG in Kawasaki disease from January 2020 to May 2023 to distinguish children with non-response to IVIG in Kawasaki disease.
Comparator	Alternative studies were not considered
Outcomes	Any clinical outcome reported by the IVIG resistance in children with KD
Timing	Before initiation of treatment with IVIG for children with KD
Setting	Patients diagnosed as showing KD in hospital or medical center/institution

KD: Kawasaki disease

#### Data extraction and critical appraisal

EndNote was used for literature management, and the title and abstract were read for primary screening and then rescreened. Two authors (WS and CY) extracted the literature content independently, and formulated and cross-checked the literature data extraction table. The extracted content included the year of publication, name of the first author, the region, the size of the sample, the number and incidence of IVIG resistance. Discrepancies were analyzed and resolved by a third experienced statistician (SN).

#### Quality evaluation

The quality of the included studies was determined using the Newcastle-Ottawa Scale (NOS), which includes eight items in three categories: selection of the population to be studied, intergroup comparability, and exposure measurement. The total possible score was 9 stars. High quality studies had ≥ 7 stars, and medium quality studies had 4–6 stars, < 4 stars indicated a low quality study. The differences in the quality evaluation process were studied by three researchers who discussed them together to reach agreement (Supplementary Table 2).

### Model building

Firstly, we selected all the risk factors which reported more than 3 times and extracted their combined OR values. Then, meta-analysis was used to calculate the pooled OR value and 95% CI of each risk factor. Using the risk factors identified in the meta-analyses, a logistic regression equation was constructed using as the coefficient of each term the result of the transformation β = ln (OR) [8]. where β is the logistic regression coefficient and OR stands for the meta-analytic odds ratio. The intercept represents the baseline value when no risk factors are present. The α constant was the natural logarithm of the ratio of resistance to IVIG: α = ln [P/ (1-P)]. The predictive model of IVIG resistance based on a logistic regression model was Logit (P) = ln [P/(1−P)] =α + β_1_ × _1_ + β_2_ × _2_ + β_3_ × _3_+. + βnXn, where Xn denotes the n^th^ risk factor and βn denotes the regression coefficient of the n^th^ risk factor. To facilitate clinical application, a risk scoring model was established based on the risk prediction model for IVIG-resistance in KD. When there was a risk factor, the regression coefficient βn of the risk factor variable was multiplied by 10, and then rounded to an integer to construct the scoring model. Thus, the IVIG-resistance risk score of KD was established, whose final score represented the sum of each influencing factor’s score. Ultimately, the probability of disease occurrence was calculated according to the total score of the risk score.

### Model validation

We selected 1007 pediatric KD cases recruited from the Children’s Hospital of Soochow University from January 2020 to June 2023 as the model validation set. Patients who had a diagnosis of complete or incomplete KD following the criteria of the AHA were included. The exclusion criteria comprised: (1) within a month before the onset of the application of corticosteroids and other immunosuppressive or blood products to children with KD; (2) Recurrent cases; (3) No fever at the time of enrollment; established infections (e.g., influenza, chickenpox, bacterial pneumonia, acute peritonitis, bacterial meningitis, and sepsis); (4) chromosomal abnormalities or severe immune diseases (e.g., immunodeficiency). The first day of illness was defined as first day that fever appeared. The baseline characteristics of patients in validation cohort show in **Supplementary Table 3**. This study was conducted in accordance with the Declaration of Helsinki and approved by the Ethics Committee of the Children’s Hospital of Soochow University (approval No. 2023CS212). Informed consent was obtained from all the participants and their parents.

### Statistical considerations

The meta-analyses were carried out using Stata 17.0 software (Stata Corp., College Station, TX, USA). To ensure the stability of the results, only the risk factors that were included in more than three studies were retained. The heterogeneity of the studies we included was determined using Q test and I^2^ statistics. Values of *p* ≤ 0.10 and I^2^ > 50% suggested there was high statistical heterogeneity among the studies. The random-effects model was used for analysis. When *p* > 0.10 and I^2^ ≤ 50%, there was no statistical heterogeneity among the studies; therefore, we chose the fixed-effects model for analysis. Egger’s regression analysis was adopted to evaluate publication bias. In addition, we performed sensitivity analyses to assess the impact of individual studies on the overall results. Thus ensuring the robustness and reliability of our findings.

Model validation data analysis was performed using the R 4.1.3 software (https://www.R-project.org/) and SPSS 27.0(IBM Corp., Armonk, NY, USA). Measurement data were expressed as median (P25-P75), and count data were expressed as percentage (%). The chi-square test was used for categorical variables, two independent t-tests were used for numerical variables conforming to a normal distribution, and the nonparametric Mann-Whitney U test was used otherwise. We used the area under the receiver operating characteristic curve (AUC) to evaluate the predictive ability and the best cut off value of the model. According to the best cut-off value, when the Youden index was at its maximum, we calculated the model’s the sensitivity and specificity. We used the HosmerLemeshow test (goodness of fit) to evaluate the calibration degree of scoring system and the decision curve analysis (DCA) to evaluate the model’s clinical practicability. Differences at *P* < 0.05 were adjudged to have statistical significance.

## Results

### Literature searching and screening

**Fig. 1:**
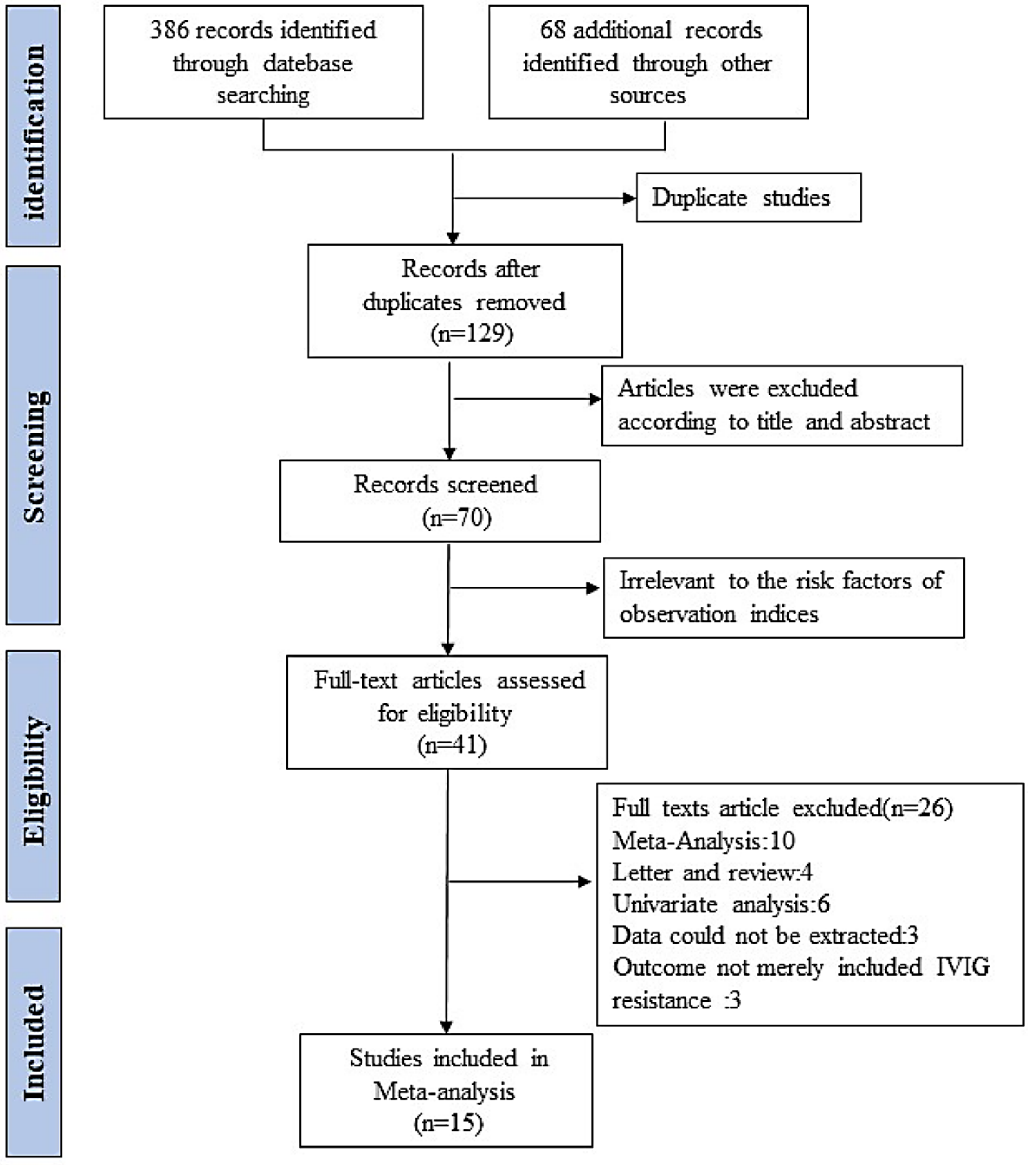
Flow chart of the Preferred Reporting Items for Systematic Reviews and Metaanalyses (PRISMA)-based selection of studies

From the three databases, we obtained 386 references, with 68 references being obtained from the follow-up, and 325 duplicate papers were eliminated. After duplicates removed, the remaining 70 full papers were screened by their title and abstract, 41 papers were excluded because they were metaanalyses (*n* = 10) or letters (*n* = 4), they used only univariate analysis (*n* = 6), data could not be extracted (*n* = 3), or the outcome did not merely include IVIG resistance (*n* = 3). Thus, 15 studies remained for analysis. These literatures had high quality (NOS score ≥ 7 points). Figure 1 shows the process used to screen the literature. Table 2 and Supplementary Table 2 show the basic characteristics of the studies and the evaluation of their quality, respectively.

**Table 2:** Characteristics of patients included in the present study

Author (year)	Country	Sample size (*n*)	IVIG resistance (*n*)	IVIG responder (*n*)	Proportion of IVIG resistance (%)	Sex (female/ male)
Muta et al. (2006) [9]	Japan	11,366	1855	9511	16.32%	4822/6544
Egami et al. (2006) [10]	Japan	320	41	279	12.81%	136/184
Uehara et al. (2008) [11]	Japan	6330	1286	5044	20.32%	2643/3687
Fu PP et al. (2013) [12]	China	1177	211	966	17.93%	431/746
Kobayashi et al. (2006) [13]	Japan	546	112	434	20.51%	231/315
Lin MT et al. (2015) [14]	China	181	22	159	12.15%	74/107
Park et al. (2013) [15]	Korea	309	30	279	9.71%	148/161
Kim et al. (2016) [16]	North America	135	22	113	16.30%	51/84
Tang et al. (2016) [17]	China	910	46	864	5.05%	326/584
Gámez-González et al. (2018) [18]	Japan	419	101	318	24.11%	175/244
Tian X et al. (2017) [19]	China	560	56	504	10.00%	210/350
Li G et al. (2021) [20]	China	1257	187	1070	14.88%	539/718
Wei M et al. (2015) [21]	China	1953	133	1820	6.81%	711/1242
Shashaani et al. (2020) [22]	Iran	363	87	276	23.97%	217/146
Sleeper et al. (2011) [23]	North America	198	27	171	13.64%	74/124

IVIG: intravenous immunoglobulin

### Results of the meta-analyses of IVIG-resistance risk factors for KD

**Fig. 2:**
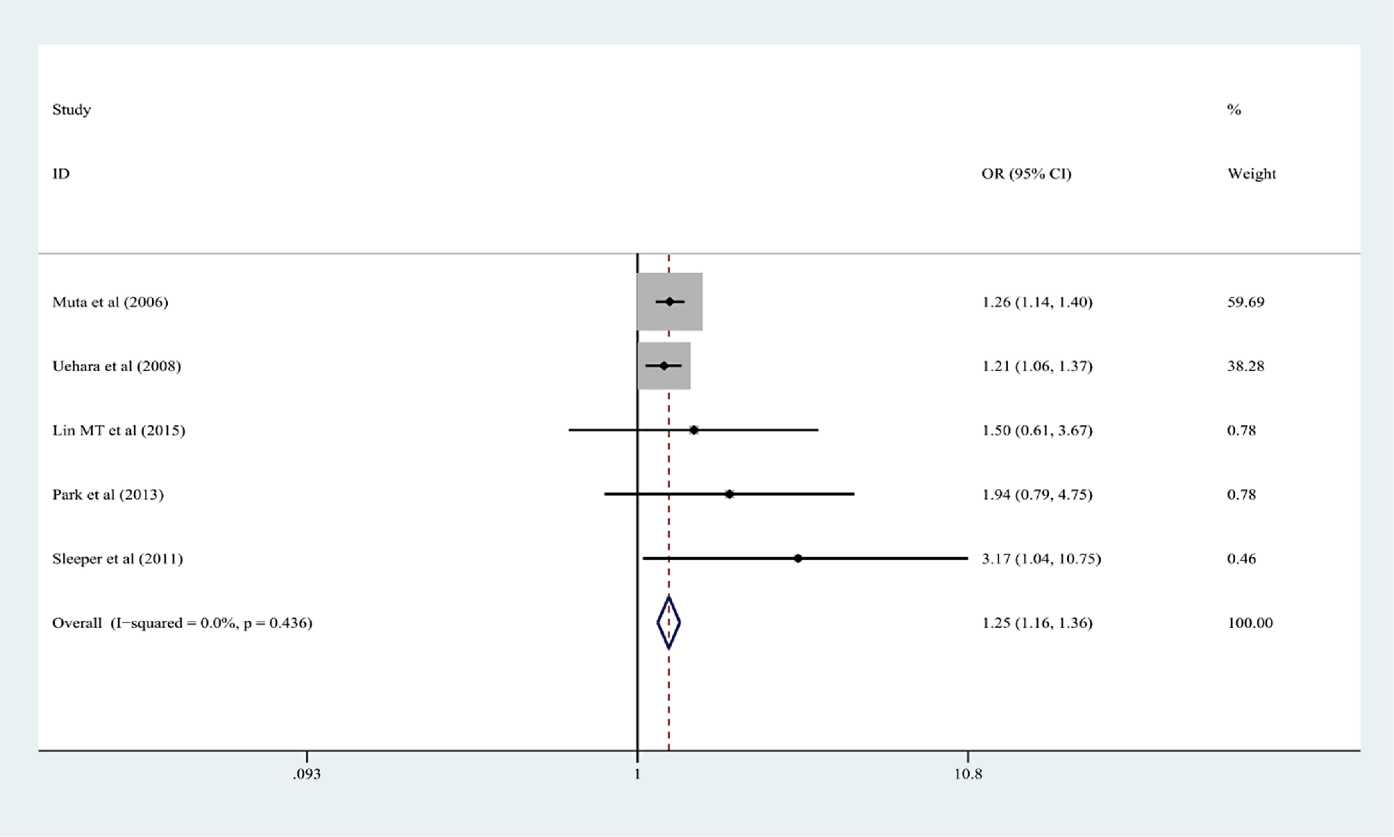
Male sex as a predictive index for intravenous immunoglobulin resistance in Kawasaki disease (KD)

**Fig. 3:**
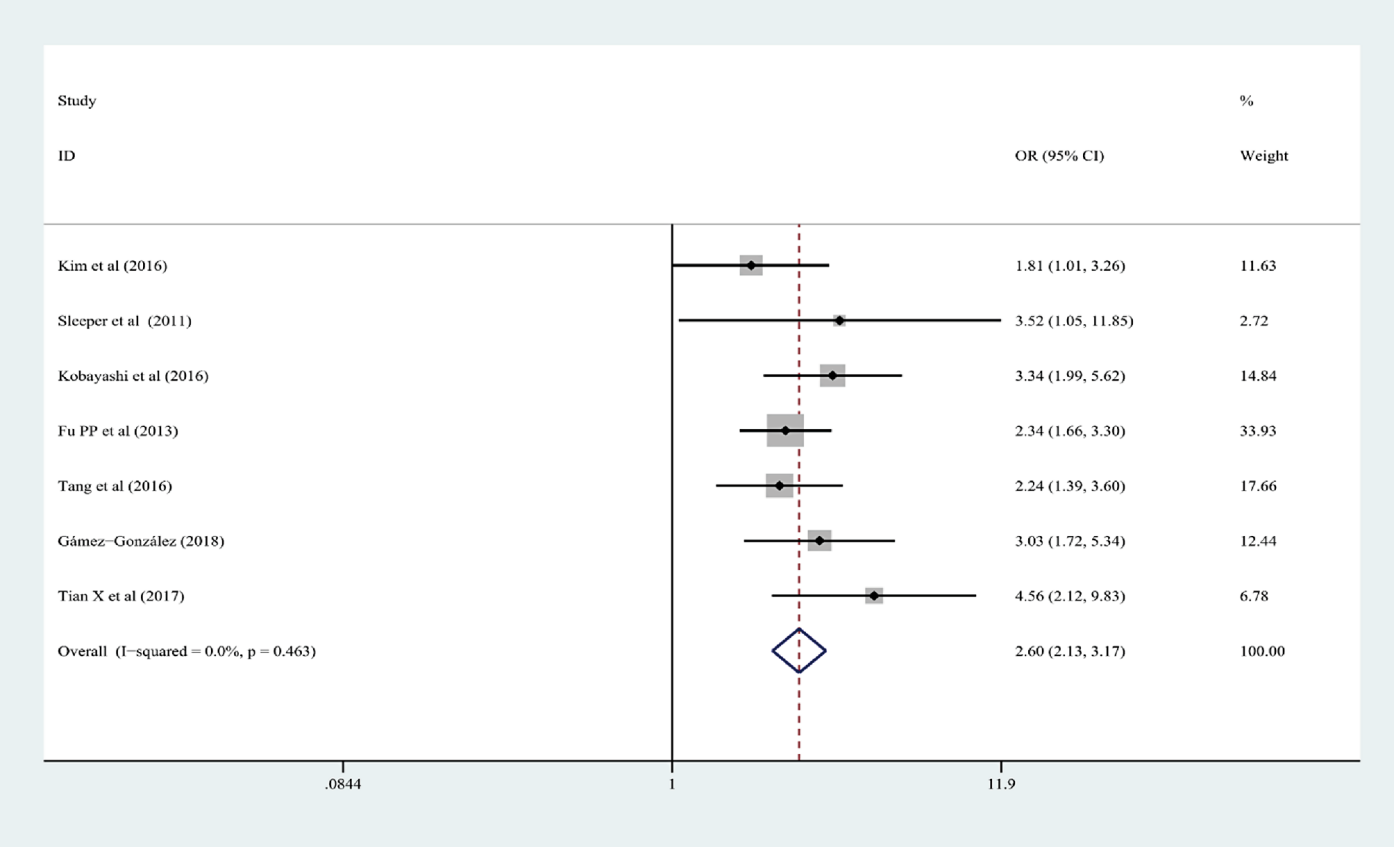
% neutrophils ≥ 80% as a predictive index for intravenous immunoglobulin resistance in Kawasaki disease (KD)

**Fig. 4:**
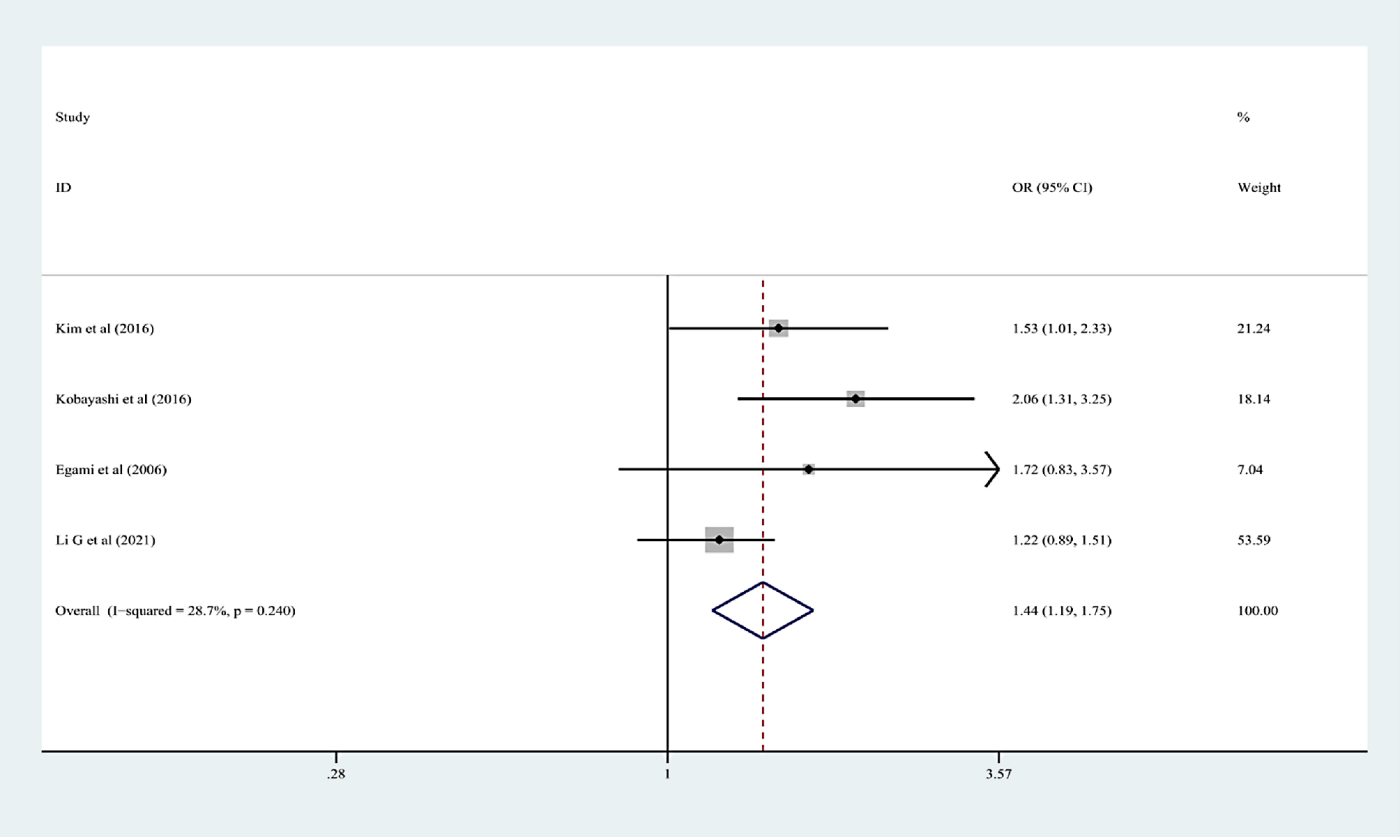
Platelet count ≤ 300 × 109/L as a predictive index for intravenous immunoglobulin resistance in Kawasaki disease (KD)

**Fig. 5:**
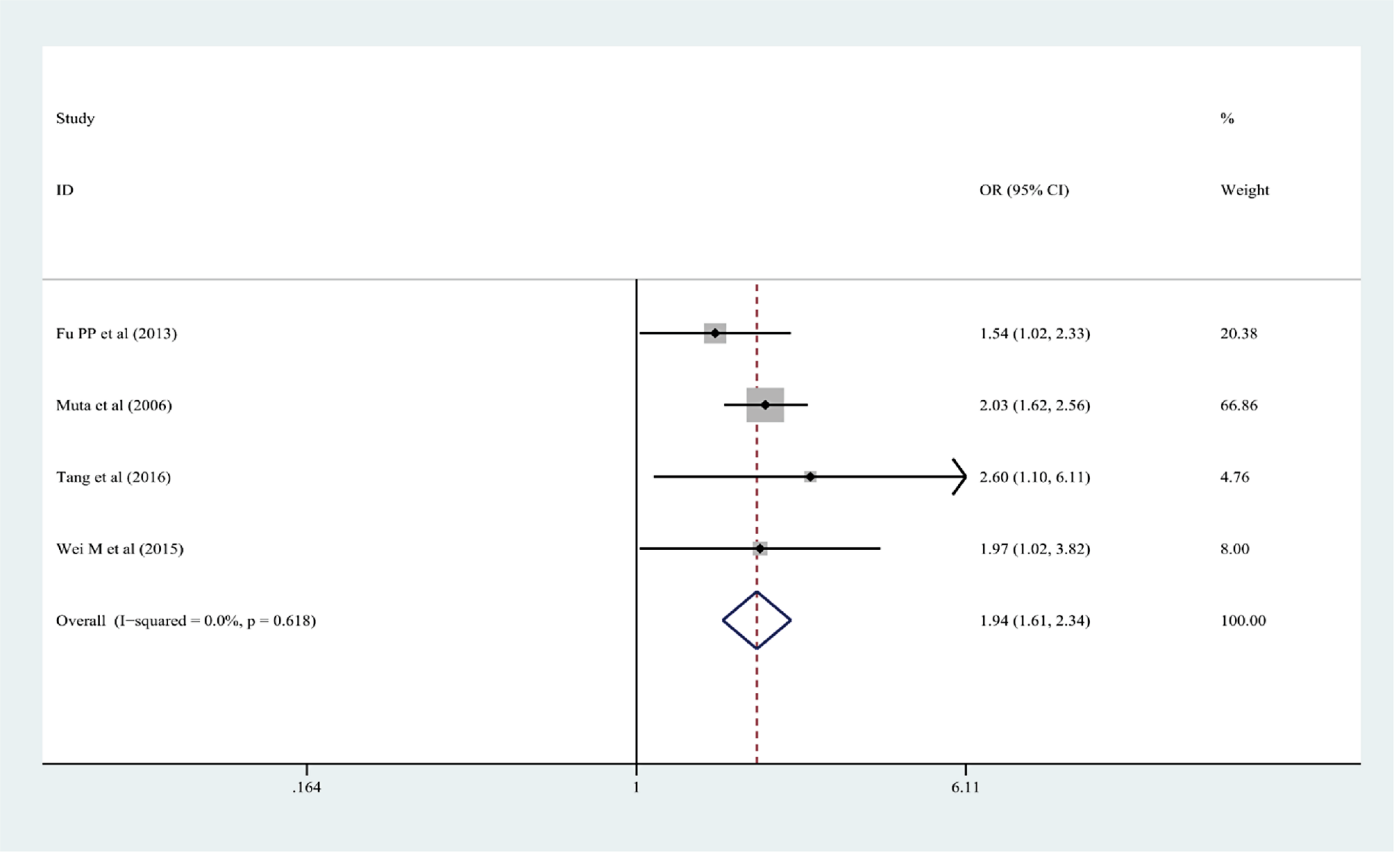
Rash as a predictive index for intravenous immunoglobulin resistance in Kawasaki disease (KD)

**Fig. 6:**
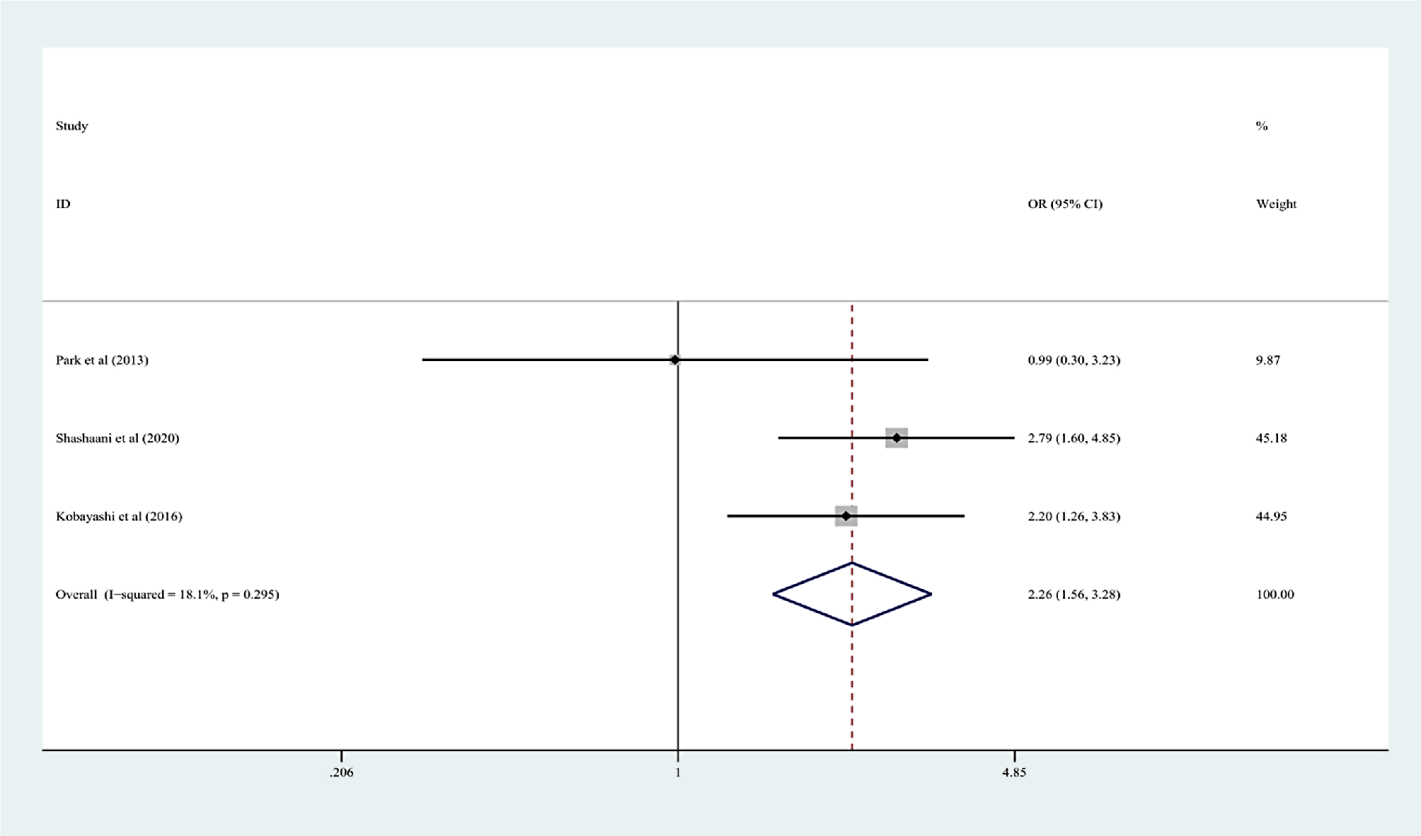
Age ≤ 12 months as a predictive index for intravenous immunoglobulin resistance in Kawasaki disease (KD)

**Fig. 7:**
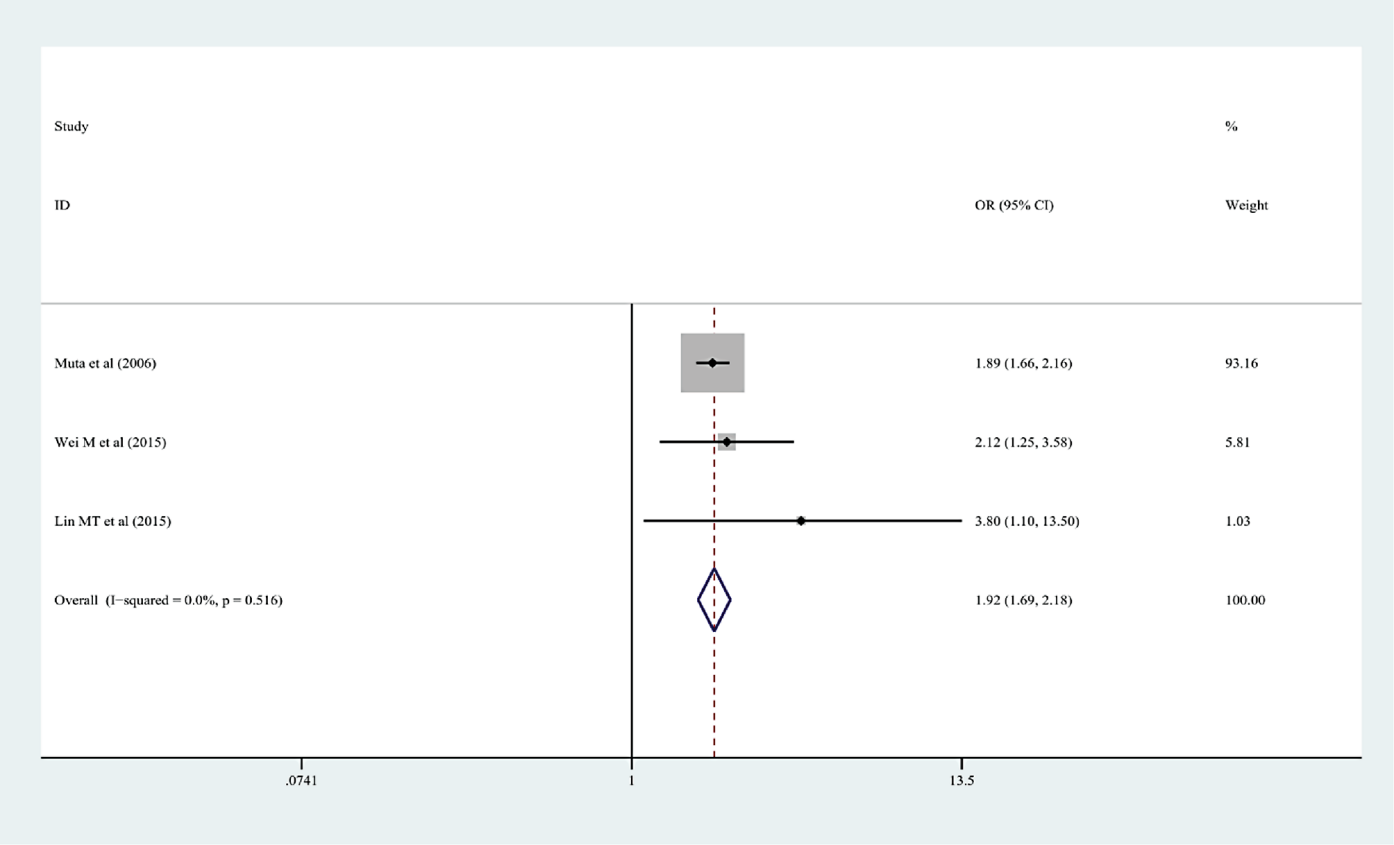
Cervical lymphadenopathy as a predictive index for intravenous immunoglobulin resistance in Kawasaki disease (KD)

In the 15 articles, 7 risk factors were reported more than 3 times, and the pooled effect size results were significant: male sex, % neutrophils ≥ 80%, platelet count ≤ 300 × 10^9^/L, rash, age ≤ 12 months, cervical lymphadenopathy, and illness day ≤ 4 (*P* < 0.05). According to the 2017 AHA guidelines, “Time to treatment ≤ 4 days” is no longer applicable to the risk factors of IVIG-unresponsive Kawasaki disease, so it was excluded from this study. Table 3; Figs. 2, 3, 4, 5, 6 and 7 show the meta-analyses and heterogeneity test results.

**Table 3:** Meta-analysis and heterogeneity test of risk factors for IVIG resistant KD

Risk factors	Number of research studies (*n*)	I^2^ (%)	*P* value	Type of Model	OR (95%CI)	Egger’s *P* value	β
Male sex	5 [9, 11, 14, 15, 23]	0	0.436	fixed-effects model	1.25 (1.16, 1.36)	0.07	2
Neutrophil ≥ 80%	7 [12, 13, 16–19, 23]	0	0.463	fixed-effects model	2.60 (2.13, 3.17)	0.251	10
Platelet count ≤ 300 × 10^9^/L	4 [10, 13, 16, 20]	28.7	0.24	fixed-effects model	1.44 (1.19, 1.75)	0.239	4
Rash	4 [9, 12, 17, 21]	0	0.618	fixed-effects model	1.94 (1.61, 2.34)	0.966	7
Age ≤ 12 months	3 [13, 15, 22]	18.1	0.295	fixed-effects model	2.26 (1.56, 3.28)	0.246	8
Cervical lymphadenopathy	3 [9, 14, 21]	0	0.516	fixed-effects model	1.92(1.69, 2.18)	0.212	7

OR: odds ratio

KD: Kawasaki disease

95%CI: 95% Confidence interval

### Sensitivity analysis and publication bias

We performed sensitivity analyses, excluding one study at a time, to investigate the stability of the results. The difference between the point and interval estimates of the combined effect size was not significant(Supplement Figs. 1, 2, 3, 4, 5 and 6).We tested the included studies for publication bias using Egger’s test, and the Egger’s test showed that the difference was not statistically significant (Table 3).

### Construction of a model to predict IVIG-resistance in KD

From January 2020 to June 2023, a total of 1007 KD children meeting with the inclusion criteria were analyzed and included in the validation set: 559 male (59%) and 408 female (41%); The mean age (months) was 22 (11,40); Platelet counts (x10^9^/L) were 325 (265, 407.5); Rash occurred in 384 cases (38%); Cervical lymphadenopathy was found in 276 cases (27%); % neutrophils were 66% (56.2, 75.55).The final Logistic risk prediction model was Logit (P) =  −1.64 + 1.25 × _1_ + 2.60 × _2_ + 1.44 × _3_ + 1.94 × _4_ + 2.26 × _5_ + 1.92 × _6_. X_1_, X_2,_ X_3,_ X_4,_ X_5,_ X_6_ stand for male sex, neutrophils ≥ 80%, platelet count ≤ 300 × 10^9^/L, rash, age ≤ 12 months, and cervical lymphadenopathy respectively. The total score of the logistic risk prediction model was 45.

### Validation of the IVIG-resistance prediction model for KD

**Fig. 8:**
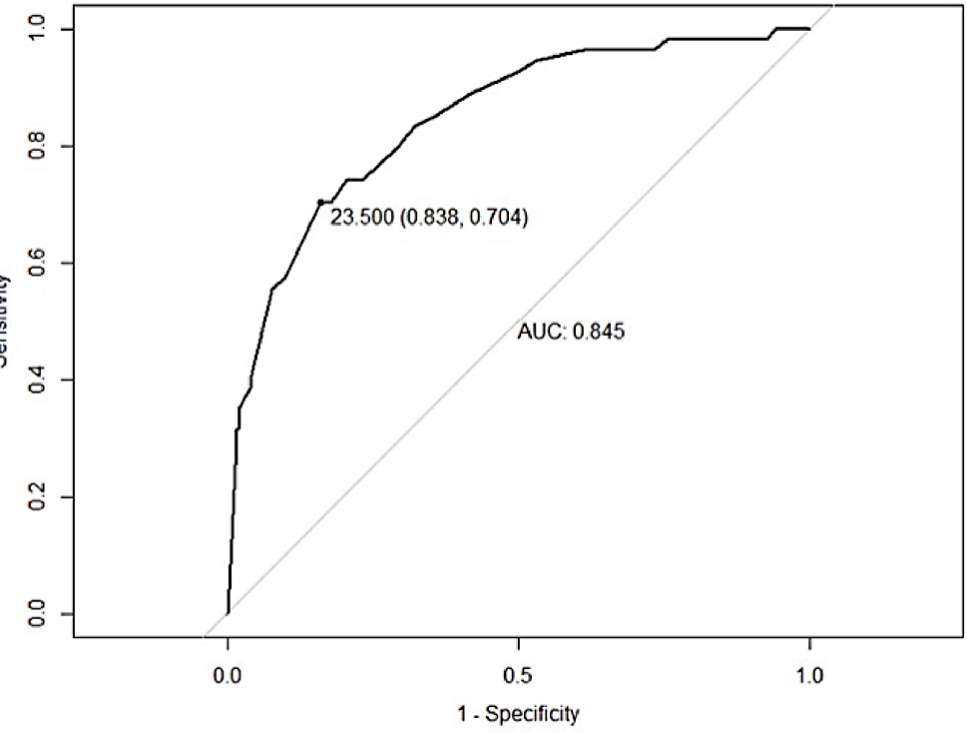
Receiver operating characteristic (ROC) curve of the risk scoring model for IVIG resistance in Kawasaki disease (KD)

**Fig. 9:**
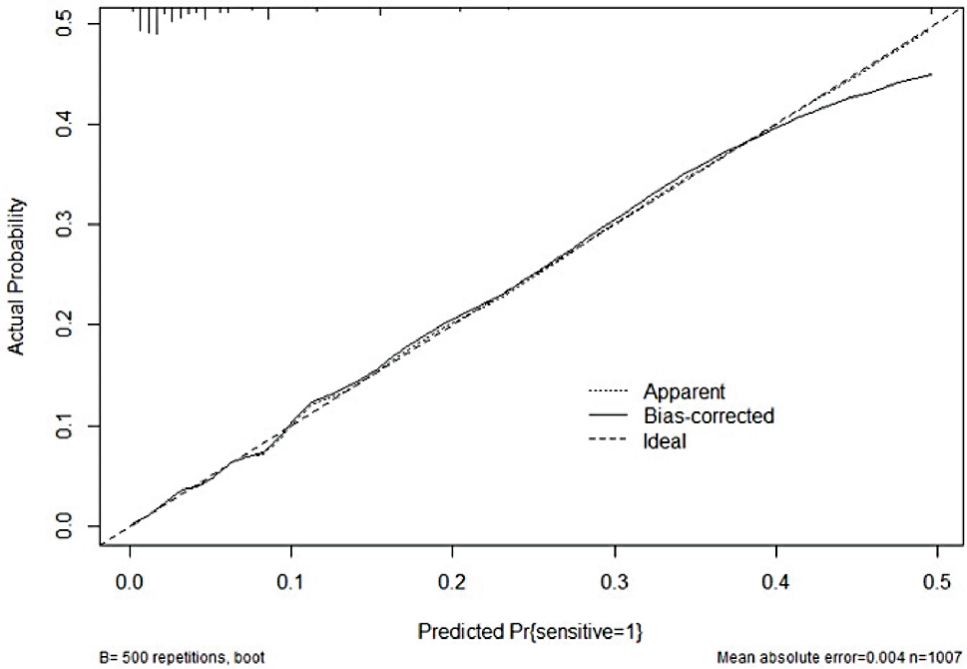
Risk scoring model calibration curves to predict IVIG resistance in Kawasaki disease (KD). The risk scoring model’s performance is shown as a dotted line. Bias correction is shown by the solid line. The models’ predictive probability in perfect agreement with the actual probability is shown as the dashed line. KD: Kawasaki disease

**Fig. 10:**
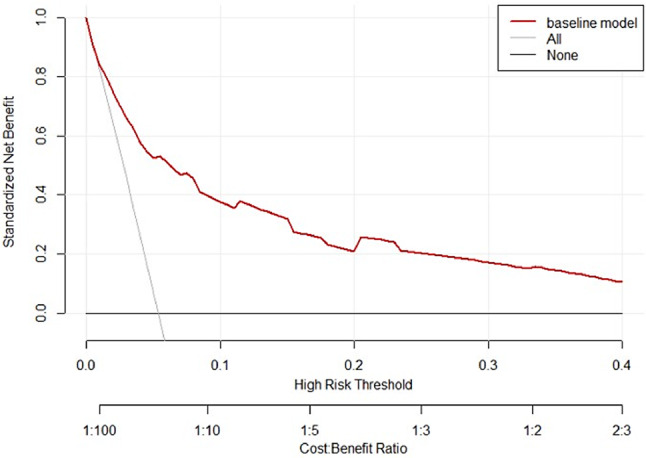
Risk prediction model’s decision curve for IVIG resistance in Kawasaki disease (KD)

The AUC of the IVIG-resistance prediction model for KD using the validation set was 0.845, and the logistic risk prediction model had a best cut-off value of 23.5 points (total score 0–45 points), the sensitivity was 83.8%, and the specificity was 70.4%, The area under the ROC curve of this prediction score was 0.845 (95% CI 0.79–0.90) (Fig. 8). The predictive accuracy between the actual probability and predicted probability was good according to the Hosmer-Lemeshow test (*P*>0.05) (Fig. 9). According to DCA, the logistic risk prediction model based on meta-analyses data had a high net benefit (Fig. 10).

## Discussion

Compared with IVIG-sensitive children, IVIG-resistant children with KD have more severe inflammation [24], a significantly increased incidence of CALs, and increased length of hospital stay and treatment costs. This study aimed to investigate IVIG-resistant KD’s clinical features and laboratory indicators, explore its related risk factors, and identify high-risk children in the early stage of the disease, which is important to allow clinicians to take effective measures and deliver individualized treatment as early as possible.

Clinical prediction models are based on mathematical models to assess the probability that a patient is in the early stage of the disease or the possibility that a certain outcome will occur in the future. Predictive models, as early warning systems, support clinical decision. Traditionally, prediction models comprised multivariate analysis regression models, decision tree algorithms, gradient boosting machine (GBM) and random forest models, which can also determine the type of disease or the prognosis of patients, However, the algorithm is complex and interpretable, and there is a “black box problem” [12, 25, 26]. To date, clinical data on pediatric KD have been used to construct models that predict IVIG resistance utilizing clinical symptoms, signs, and laboratory data. However, the small sample sizes from single centers, different methods of modeling, and a paucity of internal/external validation, have produced inconsistent outcomes, and shown poor performance in different populations [4, 27]. High-quality systematic reviews and meta-analyses are a proven method to increase statistical efficiency [28]. Herein, a systematic review and meta-analysis was used to quantitatively integrate data from multiple homogeneous studies, combine evidencebased and clinical studies, and ultimately construct a risk prediction model for resistance to IVIG. The developed model had a larger sample size, improved statistical power, and a more precise estimated effect size, thus enhancing the reliability and objectivity of the results.

The present included 15 high-quality studies, in which 4273 patients (16.2%) were identified as resistant to IVIG. The pooled risk factors were age ≤ 12 months, male sex, % neutrophils ≥ 80%, rash, cervical lymphadenopathy and platelet count ≤ 300 × 10^9^/L. These factors were consistent with the traditional risk factors of IVIG-resistant KD. Several studies found that physiological factors, including age was risk factor for IVIG-resistant KD [13, 15, 17]. Patients less than 1 year old diagnosed with KD are not only more likely to become resistant to initial IVIG therapy, but also are more likely to develop CALs [16]. In contrast, Muta et al. [9] did not consider age less 1 year old as a risk factor due to differences in methodology and study populations because physicians may prefer to treat these patients early and use high doses of IVIG to prevent complications. A previous meta-analysis of 23 casecontrol studies found that male patients (OR = 1.19, 95%CI:1.01–1.42, *P* = 0.043) and those with a rash (OR = 1.56, 95%CI: 1.20–2.02, *P* < 0.001) tended to be IVIG resistant [17]. Moreover, edema of the extremities [17], perianal change [12], and positive lymphadenopathy [14] have been regarded as risk factors for IVIG resistance; because they do not accord with standard of meta-analysis into so not included in this study. However, patients of KD with edema of the extremities, perianal change, and positive lymphadenopathy should receive more attention.

KD is an inflammatory disease associated with various pro-inflammatory factors. In children with, under the action of pro-inflammatory factors, megakaryocytes begin to multiply, thereby increasing the platelet count. Thus, the platelet count reflects the systemic inflammatory status and the activity of inflammatory pathways to a certain extent [29]. It also correlates positively with the severity of inflammation. CAA occurs significantly more frequently in the acute phase of IVIG resistance compared with that in IVIG responders, which might lead to platelet aggregation on the damaged vessel wall, thereby reducing their number in circulation. Neutrophils have vital functions in the innate immune response, and their activation is commonly seen in bacterial infections and inflammatory autoimmune diseases. IVIG is the first-line drug to treat KD, which functions by inhibiting the activated immune system and reducing inflammatory factor release. When an inflammatory response occurs, delayed neutrophil apoptosis and stimulation of stem cells by growth factors lead to increased numbers of neutrophils. The Fas antibody of IVIG can bind to the Fas antigen on neutrophils and monocytes, where it promotes cell apoptosis by activating the intracellular caspase system [30]. Currently, the percentage of neutrophils is used to predict IVIG resistance; however, the results of different studies vary greatly. The Formosa system considers % neutrophils ≥ 60% (2 points) to be sufficient to predict the risk of IVIG resistance [14]. However, Yang’s scoring system showed that % neutrophils ≥ 70% (2.5 points) could be used as a predictor of IVIG resistance before IVIG treatment [31]. However, our study is consistent with Kobayashi’s study, in which the percentage of neutrophils ≥ 80% could predict IVIG resistance [13]. The main reasons for these discrepancies are differences in sample size, region, and the affected population. In addition, there is an association between the peripheral blood neutrophil-to-lymphocyte ratio (NLR) and resistance to IVIG [32, 33]. Notably, ulinastatin, an inhibitor of neutrophil enzymes, has been used in children with KD who are unresponsive to IVIG therapy, and it is believed to improve KD clinical symptoms and prevent injury to coronary arteries.

The present meta-analyses identified IVIG treatment in the first 4 days after diagnosis of KD as an independent IVIG resistance risk factor. However, according to the 2017 AHA guidelines, the aim of KD therapy is to reduce inflammation and prevent thrombosis. Pediatric patients with KD who satisfy the diagnostic criteria should begin treatment as soon as possible, within 10 days to gain a prognosis benefit [1]. Therefore, we believe that predictors such as “Time to treatment ≤ 4 days” are no longer applicable; therefore, it was not included in our model.

Although this study is based on a combination of large amounts of evidence-based medicine data, which overcomes the bias of a single study, there are still some limitations. First, the study is based on meta-analyses to build a risk prediction model, and it is difficult to obtain original data and the logistic regression model constructed could be called “synthetic”, in the sense that the coefficients are not derived directly from raw data. Second, the prediction model of IVIG-resistance KD was verified using patient data from a single center, which only represented patients in that hospital. Further verification with a larger scope and sample size is needed to additionally refine the prediction model. Third, Given the diverse ethnic composition of the study cohort, the majority of selected articles (12 out of 15) were predominantly published from East Asia, which inevitably introduced heterogeneity in the meta-analysis methodologies. Furthermore, we utilized a single-center prospective cohort exclusively comprising Chinese individuals to validate the prediction model. thus, it is essential for this model to undergo validation in a prospective multicenter cohort involving non-Asian patients in future studies. However, with the new scoring system, all variables are easy to obtain and are convenient for clinical practice. We believe that this model has certain clinical reference value.

## Conclusions

In conclusion, the risk prediction model for IVIG resistance in KD based on 14 cohort studies consisted of 6 variables (male sex, age < 12 months, rash, cervical lymphadenopathy, % neutrophils ≥ 80%, platelet count ≤ 300 × 10^9^/L). The higher the score, the greater the possibility of requiring additional treatment. The score has a good predictive efficiency and discrimination for patients that do not respond to IVIG, and thus could provide evidence-based support for the early detection of pediatric patients with KD who are at high risk of developing serious complications. The model could be further developed for health education and to develop individualized treatment strategies.

## Electronic supplementary material

Below is the link to the electronic supplementary material.

Supplementary Material 1

## Data Availability

Data used to support the findings of this study are available from the corresponding author upon request.
