# Supplementary material for: Establishment and validation of risk prediction model to predict intravenous immunoglobulin-resistance in Kawasaki disease based on meta-analysis of 15 cohorts
Source: Ital J Pediatr. 2025 Feb 21;51:55. doi: 10.1186/s13052-025-01889-w (PMC11846198; doi:10.1186/s13052-025-01889-w)
Supplement: Supplementary file 1 — Supplementary Material 1 [file 13052_2025_1889_MOESM1_ESM.docx]

**Supplementary Table 1.** Search strategy in PubMed

| **process** | **Retrieval formula** | **Number of hits** |
| --- | --- | --- |
| **10** | ((((("Mucocutaneous Lymph Node Syndrome"[Mesh]) OR (((Kawasaki Syndrome[Title/Abstract]) OR (Lymph Node Syndrome, Mucocutaneous[Title/Abstract])) OR (Kawasaki Disease[Title/Abstract]))) AND ((((IVIG resistance[Title/Abstract]) OR (IVIG unresponsiveness[Title/Abstract])) OR (IVIG nonresponsiveness[Title/Abstract])) AND (((((predict[Title/Abstract]) OR (score[Title/Abstract])) OR (nomogram[Title/Abstract])) OR (model[Title/Abstract])) OR (risk factor[Title/Abstract])))) AND (English[Language])) AND (Journal Article[Publication Type])) AND (("2006/01"[Date - MeSH] : "2021/12"[Date - MeSH])) | 100 |
| **9** | ("2006/01"[Date – MeSH] : "2021/12"[Date - MeSH]) | 16,574,949 |
| **8** | Journal Article[Publication Type] | 33,808,014 |
| **7** | English [Language] | 31,579,137 |
| **6** | (((IVIG resistance[Title/Abstract]) OR (IVIG unresponsiveness[Title/Abstract])) OR (IVIG nonresponsiveness[Title/Abstract])) AND (((((predict[Title/Abstract]) OR (score[Title/Abstract])) OR (nomogram[Title/Abstract])) OR (model[Title/Abstract])) OR (risk factor[Title/Abstract])) | 152 |
| **5** | ((((predict[Title/Abstract]) OR (score[Title/Abstract])) OR (nomogram[Title/Abstract])) OR (model[Title/Abstract])) OR (risk factor[Title/Abstract]) | 4,014,127 |
| **4** | ((IVIG resistance [Title/Abstract]) OR (IVIG unresponsiveness [Title/Abstract])) OR (IVIG nonresponsiveness [Title/Abstract]) | 291 |
| **3** | ("Mucocutaneous Lymph Node Syndrome"[Mesh]) OR (((Kawasaki Syndrome [Title/Abstract]) OR (Lymph Node Syndrome, Mucocutaneous[Title/Abstract])) OR (Kawasaki Disease[Title/Abstract])) | 9,605 |
| **2** | ((Kawasaki Syndrome [Title/Abstract]) OR (Lymph Node Syndrome, Mucocutaneous[Title/Abstract])) OR (Kawasaki Disease[Title/Abstract]) | 8,708 |
| **1** | "Mucocutaneous Lymph Node Syndrome"[Mesh] | 7,391 |

**Supplementary table 2** Quality assessment of included studies by NOS.

| **Author (year)** | **Selection** | **Comparability** | **Outcome** | **NOS scores** |
| --- | --- | --- | --- | --- |
| **Muta et al (2006)** | ★★★ | ★★ | ★★ | **7** |
| **Egami et al (2006)** | ★★★ | ★★ | ★★ | **7** |
| **Uehara et al (2008)** | ★★★ | ★★ | ★★ | **7** |
| **Fu PP et al (2013)** | ★★★ | ★★ | ★★ | **7** |
| **Kobayashi et al (2006)** | ★★★★ | ★★ | ★★ | **8** |
| **Lin MT et al (2015)** | ★★★ | ★★ | ★★★ | **8** |
| **Park et al (2013)** | ★★★★ | ★★ | ★★ | **8** |
| **Kim et al (2016)** | ★★★ | ★★ | ★★ | **7** |
| **Tang et al (2016)** | ★★★ | ★★ | ★★ | **7** |
| **Gámez-González et al (2018)** | ★★★ | ★★ | ★★ | **8** |
| **Tian X et al (2017)** | ★★★ | ★★ | ★★ | **7** |
| **Li G et al (2021)** | ★★★ | ★★ | ★★ | **7** |
| **Wei M et al (2015)** | ★★★ | ★★ | ★★ | **7** |
| **Shashaani et al (2020)** | ★★★ | ★★ | ★★ | **7** |
| **Sleeper et al (2011)** | ★★★ | ★★ | ★★ | **7** |

**NOS：**Newcastle-Ottawa Scale

**Supplementary Figure 1:** **Sensitivity analysis of the** **“Age ≤ 12 months” association of with IVIG resistant KD**

**
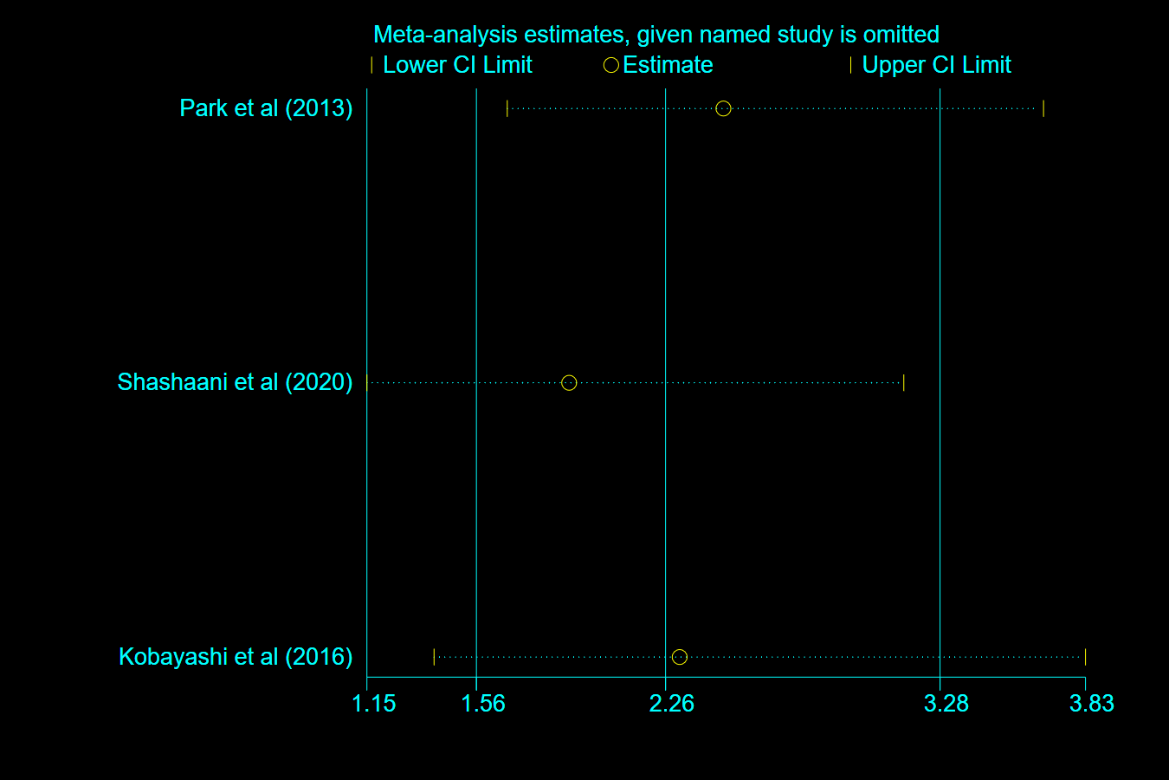
**

**Supplementary Figure 2:** **Sensitivity analysis of the “Male sex” association of with IVIG resistant KD**

**
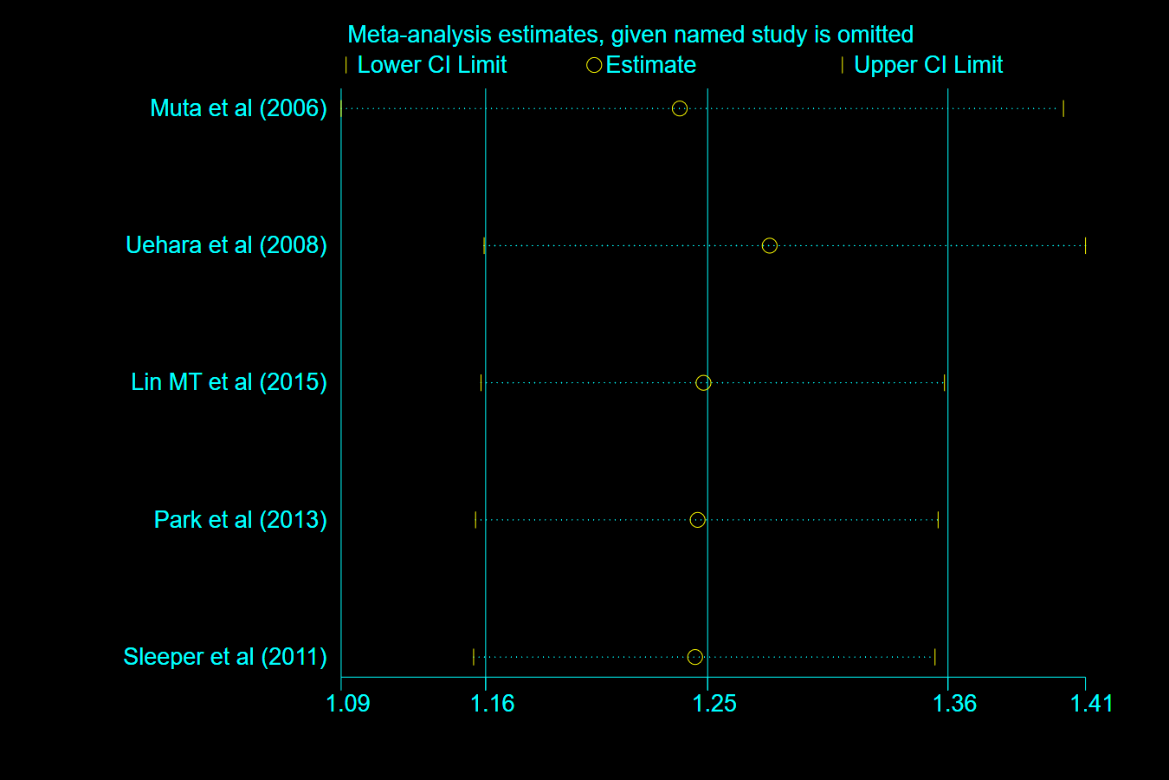
**

**Supplementary Figure 3:** **Sensitivity analysis of the “% neutrophils≥80%” association of with IVIG resistant KD**

**
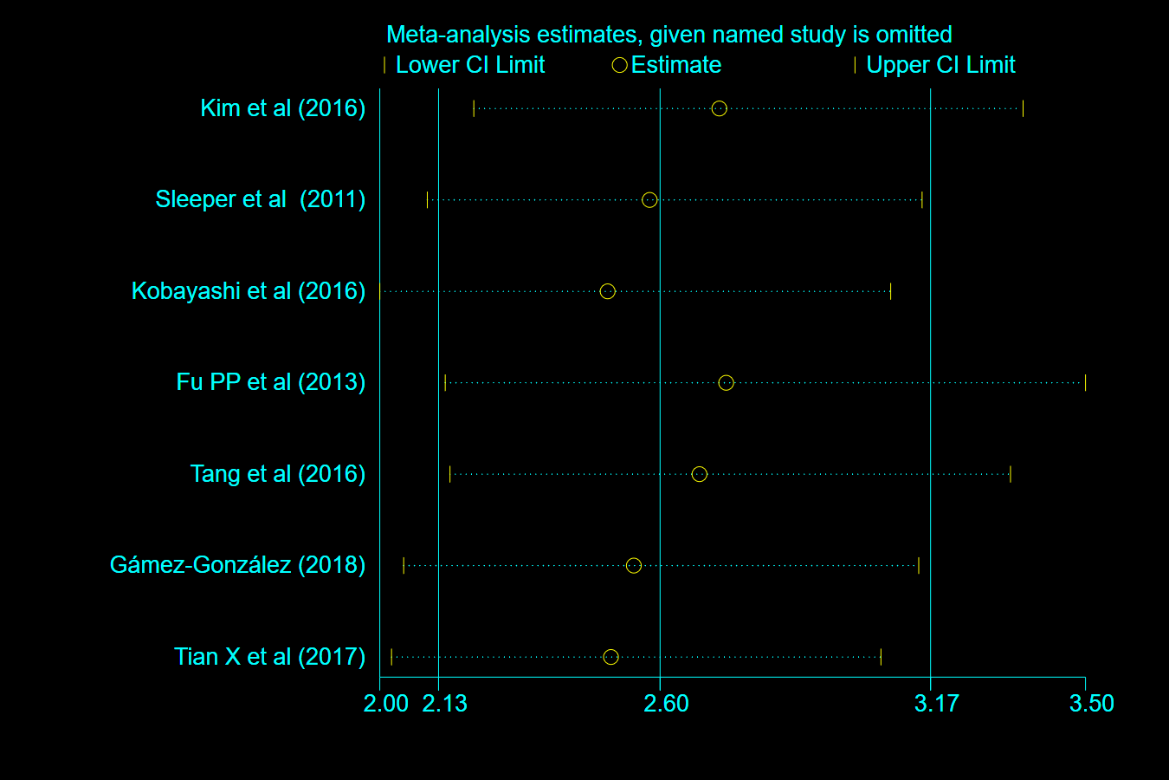
**

**Supplementary Figure 4:** **Sensitivity analysis of the “Platelet count ≤ 300 × 10^9^/L” association of with IVIG resistant KD**

**
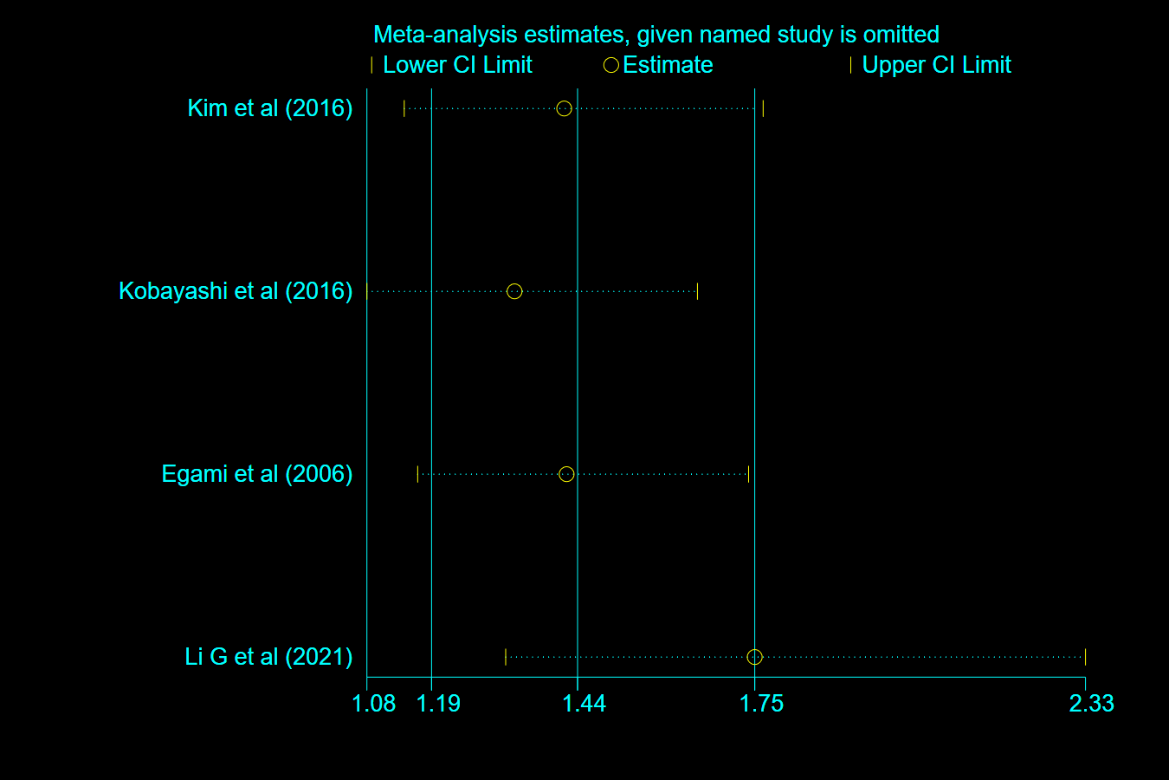
**

**Supplementary Figure 5:** **Sensitivity analysis of the** **“rash” association of with IVIG resistant KD**

**
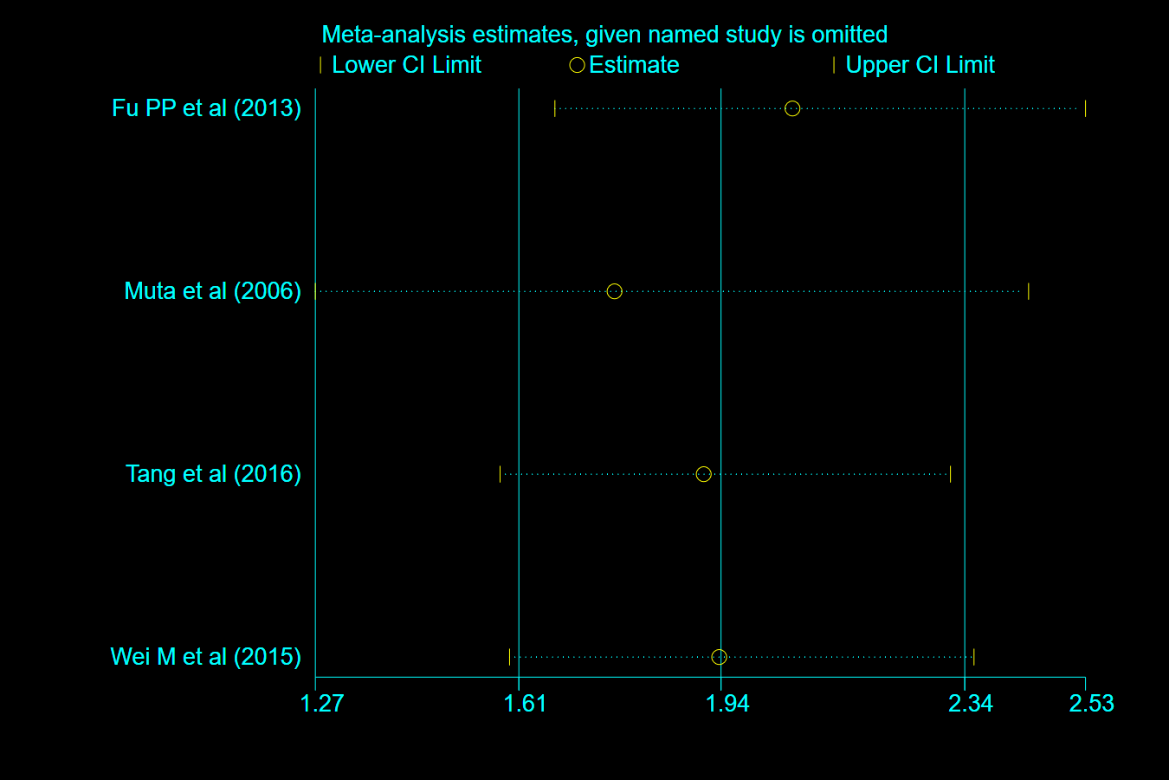
**

**Supplementary Figure 6:** **Sensitivity analysis of the “cervical lymphadenopathy” association of with IVIG resistant KD**

**
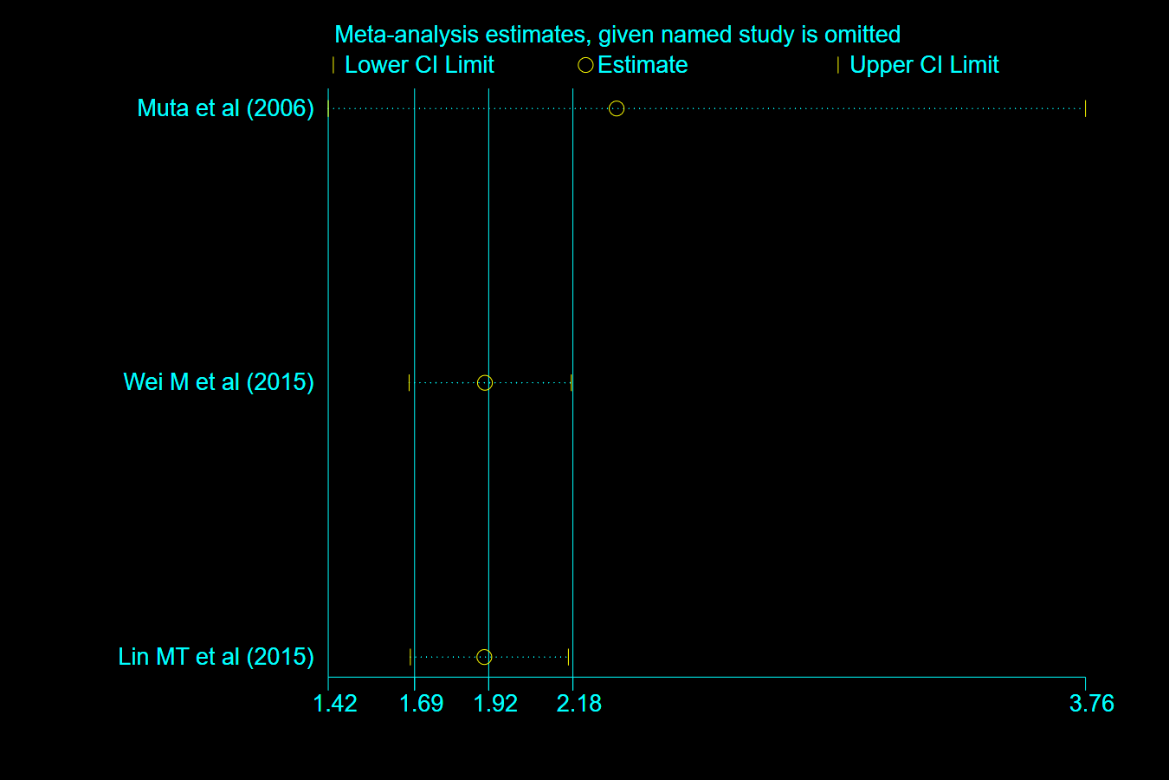
**

**Supplementary Table 3.** Baseline characteristics of patients in validation cohort

| **Variables** | **Total**  **(n=1007)** | **Responsive**  **(n=953)** | **Resistant**  **(n=54)** | **P value** |
| --- | --- | --- | --- | --- |
|  |  | **Median**  **(p25-p75)/n (%)** | **Median**  **(p25-p75)/n (%)** |  |
| Gender, n (%) |  |  |  | < 0.001 |
| Male | 599 (59) | 554 (58) | 45 (83) |  |
| Female | 408 (41) | 399 (42) | 9 (17) |  |
| Months | 22 (11, 40) | 24 (12, 41) | 8.5 (5, 13.5) | < 0.001 |
| Rash, n (%) |  |  |  | < 0.001 |
| Yes | 384 (38) | 376 (39) | 8 (15) |  |
| No | 623 (62) | 577 (61) | 46 (85) |  |
| % neutrophils | 66 (56.2, 75.55) | 65.4 (55.3, 74.9) | 75.8 (68.88, 83.18) | < 0.001 |
| Platelet, x10^9^/L | 325(265, 407.5) | 329 (266, 412) | 287.5 (227, 321.75) | < 0.001 |
| Cervical lymphadenopathy  n (%) |  |  |  | 0.001 |
| Yes | 276 (27) | 272 (29) | 4 (7) |  |
| No | 731 (73) | 681 (71) | 50 (93) |  |
